# ‘Labouring’ on the frontlines of global health research: mapping challenges experienced by frontline workers in Africa and Asia

**DOI:** 10.1080/17441692.2022.2124300

**Published:** 2022-10-02

**Authors:** Busisiwe Nkosi, Jennifer Ilo Van Nuil, Deborah Nyirenda, Primus Che Chi, Mira Leonie Schneiders

**Affiliations:** a Africa Health Research Institute, Durban, South Africa; b Institute for Global Health, University College London, London, UK; c School of Law, University of KwaZulu-Natal, Durban, South Africa; d Oxford University Clinical Research Unit, Ho Chi Minh City, Vietnam; e Malawi-Liverpool-Wellcome Trust Clinical Research Programme, Blantyre, Malawi; f KEMRI-Wellcome Trust Research Programme, Kilifi, Kenya; g Mahidol Oxford Tropical Medicine Research Unit, Faculty of Tropical Medicine, Mahidol University, Bangkok, Thailand; h Centre for Tropical Medicine & Global Health, Nuffield Department of Medicine, University of Oxford, Oxford, UK; i Socio-Ecological Health Research Unit, Institute of Tropical Medicine, Antwerp, Belgium

**Keywords:** Body work, frontline workers, everyday ethics, research ethics, Africa, Asia

## Abstract

Drawing on the reflections and discussions from a special session at the 2021 Global Health Bioethics Network summer school, this paper has summarised the key challenges faced by Frontline Workers (FWs) across research sites in Africa and Asia in performing the everyday ‘body work’ entailed in operationalising global health research. Using a ‘body work’ lens, we specifically explore and map key challenges that FWs face in Africa and Asia and the physical, social, ethical, emotional, and political labour involved in operationalising global health in these settings. The research encounter links with wider social and economic structures, and spatial dimensions and impacts on the FWs' performance and well-being. Yet, FWs' ‘body-work’ and the embedded emotions during the research encounter remain hidden and undervalued.

## 1 Introduction

### 1.1 Background

The increased attention in bioethics and frontline workers (FWs) has highlighted disparities in global health research and raised questions about FWs’ roles and responsibilities in responding to the structural concerns and needs of study participants (Khirikoekkong et al., 2020; Kingori, 2015; Nkosi et al., 2020; Steinert et al., 2021; Participants in the, 2008 Georgetown University Workshop on the Ancillary-Care Obligations of Medical Researchers Working in Developing Countries, 2008). This recognition has also highlighted the paucity of bioethics research on the hidden burdens borne by those on the ‘frontlines’ and in ‘the field’ (Gimlin, 2007). We reflect on the challenges faced by FWs through the ‘body work’ lens. While the origins of the ‘body work’ concept focused on ‘paid work on the bodies of others’ (Gimlin, 2007; Twigg et al., 2011), we use a ‘body work’ lens to include a range of activities performed by the FWs during the research encounter. These include interactions between the FWs and the participants such as recruiting, interviewing, analysing participants’ wellbeing; providing emotional and psychological support to the participants; and power relations between the FWs and participants and senior researchers. Our analysis helps to further our understanding of the impact of ‘body work’ on the FWs’ performance, and the physical, emotional and psychological toll this takes on the FWs’ well-being. FWs are at the forefront of conducting the ‘body work’ required for putting Global Health research into practice.

Studies show that the research context shapes the research encounter, and that FWs often seek to appease participants’ demands and their own conscience to make Global Health research viable (Kingori, 2013; Beard et.al., 2018, Molyneux et al., 2021; Nkosi et al., 2020). Recognising the diversity of contexts across the Global South, we explore and map key challenges facing FWs in Africa and Asia when conducting ‘body work’ required for conducting frontline research and the physical, social, ethical, emotional, and political labour involved in operationalising this work in a Global Health setting.

### 1.2 Setting and process

In this paper, we draw on the reflections and field experiences of FWs, community engagement practitioners, bioethicists, and health researchers based at the five Wellcome Trust Africa and Asia Programmes (AAP) sites, as well as participants across the sites, and from other organisations (see Table 1). All sites conduct wide-ranging multidisciplinary health research, including social science, empirical ethics, and clinical studies, and have long histories of community engagement. The research context across sites is characterised by poverty, weak health systems and limited economic and financial opportunities, often involving poor and vulnerable populations (Khirikoekkong et al., 2020; Ngwenya et al., 2020; Nkosi et al., 2020; Nyirenda et al., 2018; Steinert et al., 2021; Zakayo et al., 2020). In some sites including South Africa and Thailand the population included migrants whose legal status compounded their precarious livelihoods.

To promote and support ethical reflection, and to build capacity across the AAPs to address ethical issues in research, the Global Health Bioethics Network (GHBN) was established in 2011 as a collaborative partnership between the five AAPs, and the Ethox Centre at the University of Oxford. This paper is based on a special session at the annual GHBN Summer School of 2021, organised by the authors and focusing on challenges and interventions to support fieldworkers in the AAPs. Fifty-five GHBN members joined the virtual session chaired by BN, MLS, PCC, DN and JV, with the aim of mapping challenges faced by FWs across sites and co-producing solutions. All members were briefed about the aims of the session and at the start of the session, permission was obtained from attendees to record the plenary session and use detailed written notes to write a publication.

### 1.3 Methods

The session began with a presentation by BN and a guest presenter, PK, to situate the topic within the wider literature and to offer practical reflections. GHBN members were then split into five breakout rooms based on their geographical region in Asia and Africa to discuss key challenges experienced by FWs, existing interventions, and proposed solutions to address these. Breakout room discussions were summarised in a plenary session, offering opportunities for further joint deliberation. Based on detailed notes taken during breakout and plenary discussions, we collated, consolidated, and categorised the cross-site discussions into six main themes. In addition, we reflected upon these themes considering the contributions they make in furthering our understanding of the ‘body work’ lens over a series of subsequent virtual meetings.

## 2 Findings

### 2.1 Challenges faced by frontline workers

Below, we highlight six interconnected themes identified from the GHBN special session relating to the challenges faced by FWs in conducting Global Health research. During the session, those who worked as FWs relayed experiences that they and/or their colleagues had faced during fieldwork, while the PIs and those in managerial roles, explained experiences that had been shared to them by FWs during de-brief sessions at the respective sites. We conceptualise these experiences as the challenges resulting from the ‘body work’ conducted by FWs during research encounters with the participants. Although each theme is grouped into a main category, challenges were found to be complex, and to intersect and reinforce one another. Table 2 provides a summary of key challenges and category groupings.

### 2.2 Physical safety of frontline workers

Performance of ‘body work’ threatens integrity of FWs’ own bodies, due to the associated risks to their physical safety. GHBN members across sites highlighted some of the physical risks commonly facing FWs in the daily performance of their ‘body work’. While many communities appreciated the work of FWs, FWs tended to bear the brunt of the blame from communities when their expectations were not met. Some FWs reported being at risk or subjected to verbal, and physical abuse by communities. For instance, in South Africa, there were reports of FWs being chased away from participants’ homes, while in high crime locations, female FWs felt particularly at risk of or were threatened with sexual assault. Attacks by stray dogs, exposing FWs to risk of rabies disease were also reported risks of being in ‘the field’.

In the context of COVID-19, it was reported that visiting participants’ homes in cases where FWs lacked appropriate PPE exposed FWs to risk of infection across all sites. Additionally, some communities were wary of FWs bringing COVID-19 into their homes during research visits, which could potentially expose FWs to further physical harm. The physical risks and labour undertaken during fieldwork often overlapped with emotional labour, which will be discussed in more detail later.

### 2.3 Building relationships with research communities

The research encounter involved significant relational and emotional labour required from FWs in negotiating access to participants’ bodies-presence of mind and body to carry out the research. Members further discussed that building and maintaining relationships with research communities often brought multiple challenges to FWs. Being accepted into a community was said to take hours of relational labour to gain trust to implement data collection and maintain trust throughout the study and beyond. Relationship building may not always be immediately successful or long-lasting.

FWs sometimes faced resistance for a variety of reasons, including the disjuncture between communities’ expectations of the research, and the remit of research institutions. For example, in South Africa, participants perceived research as a service to meet their basic needs including housing, food, water and electricity, which were not provided by the government. In some instances, communities’ misconceptions of study reimbursement as payment created conflicts when ‘payments’ were perceived to be insufficient, and FWs were thus blamed for ‘stealing’ money. This not only resulted in broken trust that had been built over extended time, but also took an emotional toll on FWs.

Further challenges were emphasised for FWs doing ‘body work’ within their own communities. FWs felt they had to work particularly hard to fulfil expectations to bring resources back to their communities, even if this was beyond the scope of their role. In other instances, study methods were perceived to place participants in uncomfortable situations, for example when FWs were asked to visit a participant’s home for a research interview leaving participants feeling embarrassed by their impoverished living conditions. In one site, a FW spoke about a study with home visit interviews and while many participants were okay with researchers coming to their homes, the FW felt that a few participants were not comfortable when the FW arrived at their home. This in turn created significant emotional discomfort among FWs who felt obligated to do more after spending time listening to the participants who had spent an hour or over detailing their struggles.

COVID-19 was said to create additional burdens for FWs trying to build new research relationships with communities, due to restrictions around visiting communities to start conversations and set up collaborations. In Indonesia, this process took several weeks using remote channels, whereas before COVID-19, it would have taken considerably less time.

### 2.4 Ethical challenges

#### 2.4.1 Procedural ethics: Issues related to recruitment, informed consent, confidentiality, and trust

The complexity of conducting a truly informed consent process was a recurring theme across sites. Balancing institutional obligations, like reaching recruitment targets and ensuring that participants make informed choices about study participation created pressure and anxieties for FWs across the sites. Discussions among members suggested that FWs often rush through the informed consent forms and/or enrol participants even when they lacked comprehension about the study. In some instances, FWs’ own lack of understanding about clinical concepts undermined informed consent as they were unable to provide accurate information. This was further complicated by time pressure, and where scientific terms and ethical concepts needed to be translated, especially when equivalent expressions did not exist in the local language, thereby affecting the meaning and the concept of consent.

Impacted by social and health disparities, participants often viewed the study resources as a means for improving their lives, thereby undermining their choice to decline participation. Participants’ socio-economic status and power relationships were also seen to shape the informed consent process in important ways. For example, participants consented to performance of ‘work’ on their own bodies as a way to ensure or gain access to services and resources. For example, in South Africa, study incentives in the form of food vouchers, and phone credits were said to take precedence over informed consent. In hospitals-based studies, participants who valued hierarchical relationship structures were perceived to ‘blindly’ trust healthcare workers, making the informed consent process peripheral.

Challenges relating to maintaining participants’ confidentiality and autonomy were also highlighted. Narratives suggested that culture rather than ethical guidelines carried greater weight during fieldwork, evidenced by the importance participants placed on consulting with the immediate family about their research participation, and expecting family members to disclose information about their participation. The location of where ‘body work’ is carried out influenced FWs’ performance and well-being. When conducted in the space of a home or community, rather than a designated research institution or healthcare setting, the performance of ‘body work’ often become more complex, … as FWs negotiated and managed the participant but also other family and community members. In South Africa, enrolment of adolescents was perceived to be especially challenging for FWs, as some caregivers or parents expected FWs to advise young people about risky behaviours (e.g. substance abuse, sexual behaviours). Additionally, a lack of privacy at participants’ homes, such as when family or community members wished to join the interview, commonly compromised confidentiality, with FWs often struggling to ask them to leave. Failing to ensure participants’ confidentiality and autonomy weighed heavily on FWs, particularly when the research explored sensitive topics (e.g. infectious disease, sexual health).

#### 2.4.2 Everyday ethics: Ethical challenges and moral dilemmas of work on the frontline

Aside from the ethical issues arising around procedural aspects of research, FWs also frequently faced ethical challenges and moral dilemmas in the everyday practice of Global Health research, which were closely linked to FWs’ physical and social labour described earlier. For example, many FWs found themselves emotionally distressed when coming face-to-face with participants’ unmet basic needs (e.g. for food), or when asking sensitive questions as part of a study (e.g. about socio-economic needs, family bereavement), knowing that the study did not provide direct support to address participants’ hardships.

The heavy socio-emotional and physical labour required from FWs in trying to live up to the high expectations placed on them by research communities – who often held them in high regard and expecting them to solve their problems – was said to leave FWs feeling exhausted and ‘burned out’. Furthermore, emotional, and ethical tensions also arose for FWs whose own views, values and beliefs conflicted with the requirements of a particular study protocol. For example, drawing blood samples when a fieldworker believed this to be unethical, or promoting COVID-19 vaccination when a fieldworker was themselves vaccine hesitant among others. The moral distress caused by ethical dilemmas during fieldwork was said to be heightened among FWs who had close social and emotional ties with their research communities.

### 2.5 Ambiguity about frontline workers' roles and responsibilities

In the context of research in the Global South, ‘body work’ involves providing psychological and emotional support for the study participants, for which most FWs have not receive adequate training. While the Standard Operating Guidelines for referrals exist, there is widespread ambiguity and lack of institutional guidance about the limits of FWs’ roles and responsibilities. This ambivalence compounded many of the ethical and emotional challenges they faced. As described above, in contexts of high poverty, this lack of clarity often leads to community expectations for FWs to meet basic needs, as well as moral distress among FWs faced with deprived communities and competing community interests. The lack of support for FWs facing such recurrent structural challenges (e.g. FWs interviewing participants who are hungry) was seen to be a key area requiring further institutional support.

### 2.6 Workload, lack of support and isolation of frontline workers

Underpinned by the institutional and political contexts in which they operated, FWs experienced important structural challenges, ranging from increasing workloads, inadequate institutional support, and seasons of isolation in the discharge of their work. FWs were commonly reported to work on multiple studies for different PIs, with high demands to meet specific recruitment targets. Moreover, due to their lower hierarchical positions, FWs often felt hesitant to inform senior researchers of their workload and time constraints for fear of reprisals. Participants noted that the senior researchers are distant from the ‘bodies’ on which their work is focused. This was seen as an expression of power relations. Consequently, FWs frequently lagged in achieving their recruitment targets across different studies. FWs experience the tensions between ‘body time’ and the demands of ‘clock time’ attached to the performance of their work; the dependence of FWs’ ‘body work’ labour process on the unique and specific emotional needs of each research participants are in direct tension with the rationed and streamlined demands of ‘clock time’, which FWs find themselves bound by (Davies, 1994; Twigg et al., 2011). In this prevailing situation, many FWs felt overwhelmed and inadequately supported by their PIs and institutions as support systems (e.g. debrief sessions) tended to focus on the research process and neglected FWs’ well-being. The perceived lack of institutional support felt by FWs may equally engender emotional distress, while also interlinking with and generating ethical challenges (e.g. poor administration of the informed consent process).

Additionally, members discussed that FWs tended to work in silos within the same institution, without an enabling environment to share their experiences (negative and positive) and lessons learnt during their work. This was further compounded by the institutional requirements and good research practice to maintain participants’ confidentiality in the process of data collection. These prevailing institutional challenges were said to further exacerbate the precariousness of FWs’ work environments by compounding the social, emotional, and ethical challenges they faced.

## 3 Discussion

The ‘body work’ nexus provides an opportunity for examining how the social and economic contexts, and power relations impact the FWs’ performance during the research encounter. FWs experience enact hidden burdens and various forms of labour while working on the ‘frontline’ of Global Health research (Kingori, 2013; Molyneux et al., 2021). These ‘labours’ are significant and span across physical; social and relational; ethical and moral; emotional and psychological; and political, institutional, and structural boundaries. Our reflections highlight the following. Firstly, the performance of ‘body work’ threatens the integrity of FWs’ own bodies, due to the physical safety risks associated with the everyday work on the frontlines of Global Health research. Secondly, FWs undertake significant relational and emotional labour in ensuring participants’ well-being during the research process. Additionally, owed to the challenges surrounding ambiguity about FWs’ roles and responsibilities in Global South research contexts, the ‘body work’ conducted by FWs extends to support basic needs and the psychological and emotional and wellbeing of their participants domains of work for which most FWs do not receive adequate training or support (Beard et.al., 2018, Nkosi et al., 2020; Steinert et al., 2021).

Thirdly, the challenges related to FWs’ role in safeguarding the procedural aspects of ethical research (e.g. informed consent and confidentiality) as well as the challenges related to high workloads highlight the important temporal dimensions of FWs’ ‘body work’ in Global South contexts. For example, FWs experienced tensions between ‘body time’ of their participants and the demands of ‘clock time’ in their everyday work, such as when ascertaining informed consent, or when needing to attend to the unique and specific emotional needs of their participants. Such labour appears in direct conflict with the rationed and streamlined demands of ‘clock time’, which FWs find themselves bound by under the demands of ambitious research timelines and recruitment targets. Similarly, others have argued that ‘the dependence of the body work labour process on bodily needs makes it difficult to rationalise or speed up’ (Steinert et al., 2021; Twigg et al., 2011).

Additionally, the reflections emphasise the importance of the spatial dimension of conducting ‘body work’ in Global South contexts, in that they highlight the significance of the location where such work is carried out. For example, when conducted in a home or community setting, rather than a designated research institution or healthcare setting, the performance of ‘body work’ often becomes more complex, as negotiating and managing the research process not only involves the FW and participant, but also the wider family and/or community members. Finally, the spatial separation and physical distance between FWs conducting ‘body work’ on the frontlines and PIs and research institutions who solicit such research but are distant from the ‘bodies’ on which their work is focused on, can be seen to be expression and representation of the underlying hierarchies and power relations underpinning the research encounter itself (Twigg et al., 2011).

Our reflections expand on previous work by illustrating how FWs are largely left unsupported while navigating everyday ethical challenges in their work (Kingori, 2013; Nkosi et al., 2020; Steinert et al., 2021). As we previously demonstrated, in contexts of inequalities, FWs experience empathy and sympathy for the participants due to their inability to meet participants’ basic and health needs (Beard etal., 2018). Across sites, everyday ethical challenges arising beyond the procedural aspects of research created significant emotional burdens and moral distress for FWs (Twigg et al., 2011).

While the discussions focused mainly on mapping challenges experienced by FWs, there was consensus that potential solutions must be multifaceted to address the structural problems underlying the challenges faced by FWs in operationalising global health. Furthermore, members emphasised the importance of strengthening the institutional systems already in place, for example by adapting the debrief sessions to address and support the embodied experiences facing FWs instead of mainly focusing on the research process. In Kenya, an institutionalised model ‘ethics reflections’, has been developed to support and build FWs’ capacities to respond to the ethical dilemmas (Molyneux et al., 2021).

Finally, despite the increased attention and consideration being paid to the challenges faced by FWs across the AAPs research sites, many issues still surfaced in our members’ discussions. As illustrated, these issues are complex and require multi-faceted approaches and solutions to strengthen and support the work of FWs.

## 4 Conclusion

We summarised the key challenges faced by Frontline Workers (FWs) across research sites in Africa and Asia in performing the everyday ‘body work’ entailed in operationalising global health research. Our findings show that FWs’ ‘body work’ and the embedded emotions during the research encounter remain hidden and undervalued. More research is therefore needed to explore solutions to the challenges facing FWs operating in Global South contexts.

## Figures and Tables

**Table 1:** Wellcome Trust Africa and Asia Programmes (AAP) sites, including the number of participants, broken down by region and position.

Site	Region	Participants (n)	Position
Africa Health Research Institute (AHRI), South Africa	Africa	16	Community Engagement staff = 4 Frontline researchers = 4 Principal Investigators = 6 Managers = 2
KEMRI-Wellcome Trust Research Programme, Kenya		18	Principal Investigators = 7 PhD students = 2 Frontline Workers = 4 Community Engagement staff = 5
Malawi-Liverpool-Wellcome Trust Clinical Research Programme (MLW), Malawi		17	Principal Investigators = 1 Post doctoral researchers = 3 PhD students = 3 Masters students = 2 Community Engagement staff = 3 Frontline Workers = 4
Mahidol Oxford Research Unit (MORU), Thailand, Myanmar	Asia	12	Positions unknown
Oxford University Clinical Research Unit (OUCRU), Vietnam, Nepal & Eijkman-Oxford Clinical Research Unit (EOCRU), Indonesia		14	Frontline Workers = 11 Principal Investigators = 2 Managers = 1
Ethox Centre	UK	10	Principal Investigators = 4 Frontline Workers = 6
Other (not from AAPs)	Global	11	Positions Unknown

**Table 2:** Examples of challenges facing frontline workers (FWs) in global health across five Wellcome Trust Africa and Asia Programmes (AAPs).

Category of labour undertaken by FWs	Main theme of challenge	Example of challenges facing FWs across the AAPs	Overlapping categories
PHYSICAL	Physical safety of FWs	• Risk of abuse and assault (especially among female FWs)	• Emotional
		• Risk of attack from stray dogs (risk of rabies disease)	• Emotional
		• *Additional challenges due to COVID*: Heightened risk of infection with COVID-19 among FWs visiting participants' homes	• Emotional
SOCIAL/relational	Relationships with research communities	• Being accepted into a community/building trust/relationship with community (relational work to gain acceptance)	• Emotional
		• Facing resistance and backlash from community and taking the blame when there are problems with the research	• Emotional
		• Accused of stealing money from community when there is a perception of inadequate 'payment' (compensation) for research study participation	• Emotional
		• Participants feel uncomfortable to have FWs in their home for an interview because they live in poverty, while study protocol requires FWs spend time in participants' homes	• Ethical/Political
		• FWs who are from the same community as study participants feel they need to work extra hard to bring benefits to their communities	• Ethical/Emotional
		• *Additional challenges due to COVID*: Difficulties building trust remotely when in person visits of community are restricted	• Political
ETHICAL/moral	Procedural ethics: Issues related to recruitment, informed consent, confidentiality, and trust	• FWs need to rush the consent process to meet demanding recruitment targets, aware that participants may lack understanding about study and informed consent may be undermined	• Political/Physical
		• FWs do not always understand study < protocols and just hand out info sheets, worried that community won't have confidence in them if they can't answer questions about the study	• Social/Political
Category of labour undertaken by FWs	Main theme of challenge	Example of challenges facing FWs across the AAPs	Overlapping categories
		• Participants join research because of hierarchal structures of relationships and trust in healthcare workers and research institutions, compromising informed consent	• Social/Political
		• Diversity of ethnicity at field sites cause challenges for translation and interpretation of study protocols and ethical terms, particularly when translators are not part of study team	• Social
		• FWs struggle to ensure privacy and confidentiality of participants as required by the study protocol in community-based studies when family and community members listen in during data collection	• Social
ETHICAL/moral & EMOTIONAL/psychological	Everyday ethics: ethical challenges and moral dilemmas	• FWs' personal values/world views conflict with the views they need to promote as part of a study (e.g. around COVID vaccination)	• Social
		• FWs' moral distress and discomfort when needing to ask difficult or sensitive questions when they know study won't help to address these issues	• Social/Political
		• Community expectations that FWs will solve community problems leads to high stress and burnout	• Social/Physical
ETHICAL/moral & POLITICAL/institutional/structural	Ambiguity surrounding fieldworkers' roles and responsibilities	• FWs face moral distress when working in contexts of deprivation and poverty, when research studies do not align with community interests or meet basic needs (e.g. for food)	• Emotional/Social
		• Communities expect FWs to provide basic necessities, but research protocols do not provide for this, putting FWs in uncomfortable positions (e.g. interviewing someone who is hungry, but being uncertain if they should share the lunchbox with them)	• Emotional/Social
POLITICAL/institutional/structural	Workload, lack of support and isolation of fieldworkers	• FWs work in silos and don't share their experiences/solutions together, partly due to concerns about confidentiality of research	• Social/Emotional
Category of labour undertaken by FWs	Main theme of challenge	Example of challenges facing FWs across the AAPs	Overlapping categories
		• FWs face significant workload and time pressure to recruit high sample sizes, often while working on multiple studies with different PIs, resulting in stress and burn out	• Physical/Emotional
		• FWs don't feel supported by PIs who do not understand the context of everyday fieldwork	• Emotional
